# The status of active after-school clubs among primary school children in England (UK) after the COVD-19 lockdowns: implications for policy and practice

**DOI:** 10.1186/s12966-023-01499-x

**Published:** 2023-10-05

**Authors:** Robert Walker, Ruth Salway, Danielle House, Lydia Emm-Collison, Katie Breheny, Kate Sansum, Sarah Churchward, Joanna G Williams, Frank de Vocht, William Hollingworth, Russell Jago

**Affiliations:** 1 Centre for Exercise, Nutrition & Health Sciences, School for Policy Studies, University of Bristol, Bristol, BS8 ITZ UK; 2 Population Health Sciences, Bristol Medical School, University of Bristol, Bristol, BS8 2PS UK; 3 The National Institute for Health Research, Applied Research Collaboration West (NIHR ARC West), University Hospitals Bristol and Weston NHS Foundation Trust and University of Bristol, Bristol, BS1 2NT UK; 4 Independent public member of the project team, Bristol, UK; 5 Communities and Public Health, Bristol City Council, Bristol, UK; 6 NIHR Bristol Biomedical Research Centre, University Hospitals Bristol and Weston NHS Foundation Trust and University of Bristol, Bristol, BS8 2BN UK

**Keywords:** Physical activity, Sport, Exercise, Cost of living crisis, Spending, Club provision

## Abstract

**Background:**

Children’s physical activity in England is more dependent on active clubs after the COVID-19 pandemic. However, it is unclear how the COVID-19 pandemic and related cost-of-living crisis have impacted on active club participation, costs and provision. This mixed-methods natural experiment explored school-based and community-based active clubs after lockdowns, using a unique combination of data sources to highlight implications for policy and practice post-COVID-19.

**Methods:**

Cross-sectional questionnaire data on school and community active clubs were collected from 10-11-year-old children pre-COVID-19 in 2017-18 (N = 1,296; 50 schools), in 2021 (N = 393; 23 schools), and 2022 (N = 463; 27 schools). Club participation and attendance frequency were modelled using logistic and Poisson mixed effects models, adjusted for child age, gender and household education. In 2021 and 2022, parents reported expenditure on community-based clubs and schools provided data on school-based club provision, with data summarised descriptively. Qualitative data were collected in 2021 and 2022, with one-to-one interviews with school staff (N = 18) and parents (N = 43), and twelve child focus groups (N = 92), and analysed using the framework method.

**Results:**

School-based active club participation was higher in 2022 compared to pre-pandemic (50% /43%), while community-based club participation was lower (74%/80%). Children attended 0.3 fewer clubs per week. Those from lower education households were less likely to participate in both types of active clubs, and girls less likely to attend community clubs. In 2022, the median cost of community and school club sessions were £6.67 and £3.88 respectively, with 52% of school-based clubs free to parents. Schools offered an average of 3.4 active clubs per week for 10-11-year-olds in 2022, with 34% partly/wholly subsidised. Qualitative analysis highlighted the impact of the cost-of-living crisis and COVID-19 pandemic on family resources, encouraging a shift to more affordable and convenient school-based active clubs, which negatively impacted the community-based active club environment. However, many schools struggled to meet this increased demand.

**Conclusions:**

Findings emphasise the importance for policymakers to support schools to meet increased demand for clubs and community clubs to increase affordable and convenient physical activity opportunities. Targeted support is needed to prevent socioeconomic and gender inequalities.

**Supplementary Information:**

The online version contains supplementary material available at 10.1186/s12966-023-01499-x.

## Background

Physical activity among children is positively associated with many physical and psychological factors that promote wellbeing [1–4]. The World Health Organization and UK Chief Medical Officers recommend that children should accumulate an average of at least an hour of moderate-to-vigorous intensity physical activity (MVPA) per day [5–7]. Yet, only 41% of 10-11-year-old children in the UK met these guidelines prior to the pandemic [8].

The COVID-19 pandemic significantly impacted children’s physical activity around the globe, as many countries implemented lockdowns and restrictions on movement and social/leisure activities [9–12]. To evaluate the impact of the pandemic on children’s physical activity in England, we developed the Active-6 project, adopting a repeated cross-sectional natural experiment design which compared an existing pre-pandemic dataset of children aged 10–11 years’ physical activity levels collected in 2017/18 to new data collected during/after the pandemic [12–19]. Findings from this project suggest that, after a short-term drop in 2021, average children’s MVPA had recovered to pre-pandemic levels by mid-2022, although weekday sedentary time remained elevated by around 13 min per day [15, 20]. Despite this recovery, it is important to highlight that the majority of children (59%) in the study still did not meet physical activity guidelines [15]. We also observed a greater dependence on structured activities (that is, those that are organised and planned into the family’s schedule, e.g. sport or exercise clubs) rather than unstructured activities (those that are spontaneous and unplanned, e.g. active play), which we have termed the new normal for children’s physical activity [16]. This finding emphasises the greater role of active clubs in children’s post-pandemic physical activity behaviour, conceptualised as structured, adult-led sport or exercise clubs (e.g. football, athletics, dance, martial arts).

Children’s participation in active clubs is important for increasing levels of physical activity [21–23], and constitute one of key indicators of the report card on physical activity for children and youth from the Active Healthy Kids global alliance [24]. This report card aims to achieve a comprehensive understanding of global physical activity and its related indicators using scores ranging from F to A+. England received a grade D for children’s participation in organised sport and physical activity, which was estimated at 33% of children and adolescents, and received an overall average grade of C- [24]. Active clubs therefore present an area of opportunity to increase levels of physical activity among children in England.

Active clubs in England can be broadly categorised into school- and community-based clubs. The government PE and Sport premium provides schools with funding to improve their physical activity provision [25], allowing children to participate in active clubs at their school often at a discounted/reduced cost. This means that active clubs based in the community tend to be relatively more expensive and provided by private organisations. However, to date, research has not evaluated the costs of active clubs, or how provision may have changed post-pandemic. It is important that we understand the active club environment if we are to promote physical activity among diverse groups of children in the new normal in which they live.

Girls and children from lower socioeconomic households are two groups who experience increased challenges to participating in active clubs, which may have been further exacerbated during the pandemic [16]. Moreover, in the context of high inflation for food and energy on an international scale (referred to as the “cost-of-living crisis” in the UK), it is possible that active club participation and provision has been impacted and pre-existing inequalities exacerbated by economic challenges.

It is therefore important to understand post-COVID-19 levels of active club provision, participation and the associated costs in England. Using a rich combination of mixed-methods data collected from parents, children, and schools, we explored how active club participation, provision, and costs have changed since the COVID-19 lockdowns, and highlight key implications for policy and practice.

## Methods

**Fig. 1:**
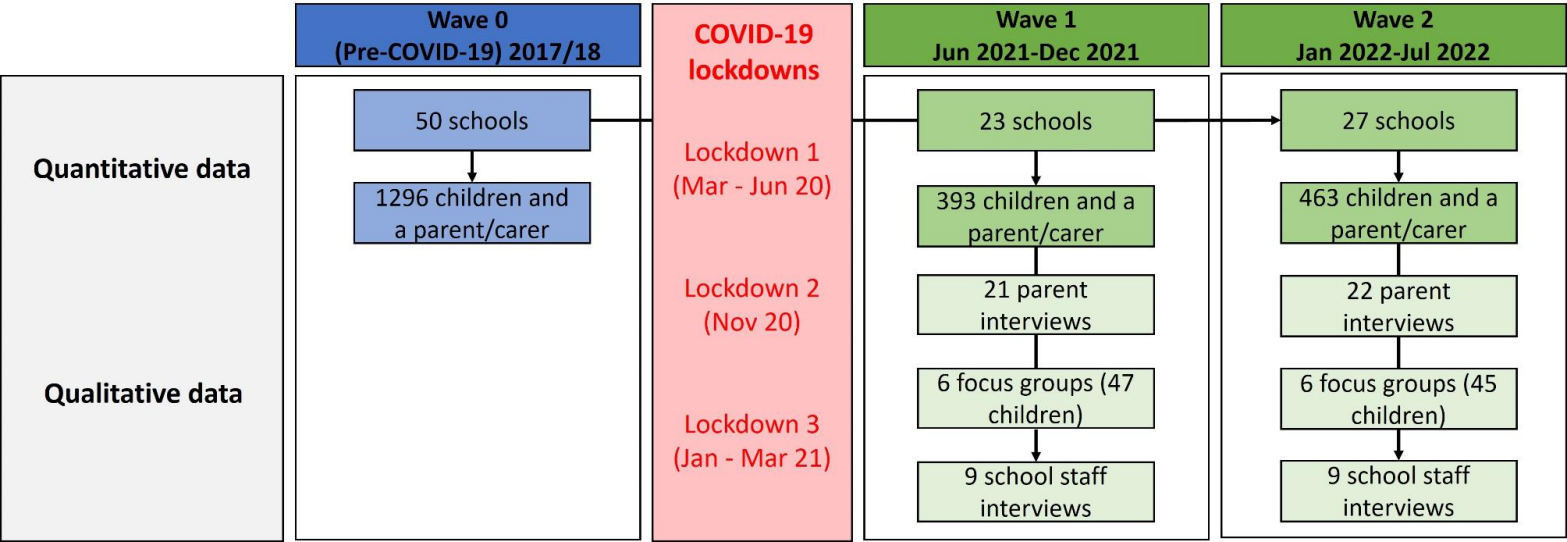
Data collection process

A summary of the data used in this paper is provided in Fig. 1. We employed a partially mixed, concurrent, equal status mixed-methods design, whereby qualitative and quantitative data analysis occurred in parallel but findings were not combined until the final interpretation [26]. This approach allowed us to explore this topic from different angles and draw upon the strengths of each method. This study was based in critical realism [27] where we aimed to qualitatively identify the structures and mechanisms that explained the changes in observed events identified in quantitative analyses, and combine this information to deepen findings.

The study received ethical approval from the School of Policy Studies Ethics Committee at the University of Bristol, UK (Ref SPSREC/20–21/150), and all participants provided informed consent/assent for both qualitative and quantitative aspects of this study.

### Quantitative methods

#### Participants and procedure

Active-6 quantitative methods have been published in detail elsewhere [12, 15]. In short, B-Proact1v was a five-year longitudinal study of children and their parents/carers. The final phase in 2017/18 when the children were aged 10–11 years, hereafter referred to as Wave 0, included 1296 children and one parent/carer from 50 state schools. In Active-6, the same 50 schools were invited to take part in two additional waves, with 23 schools (46%) participating in Wave 1 between June and December 2021 and 27 (54%) in Wave 2 between January and July 2022 (22 schools took part in both waves). Quantitative data were collected from children aged 10–11 and one parent/carer at each wave, with a total of 393 child-parent dyads in Wave 1 (38% of those eligible) and 463 children in Wave 2 (35%).

#### Data

Children and parents completed online/written questionnaires and a representative from each school provided data on school-based active clubs.

##### Sociodemographic data

Parents/carers reported child and parent gender, child date of birth, parent age group and highest household education qualification. In Waves 1 and 2 only, parents also reported perceived financial difficulties using the family economic strain scale (FESS), a validated 13-item measure using a 5-point Likert scale [28]. Items were summed to give a total FESS score, with higher scores reflecting greater economic strain [28]. Participants were categorised by FESS quartile, calculated across both waves combined.

The research team also collected information related to school characteristics. Index of Multiple Deprivation (IMD) [29] was collected using the school postcode, with lower scores indicating a greater level of deprivation. Percentage of pupils receiving a free school meal, a government initiative to supply disadvantaged children with a nutritious meal at school [30], was collected by the research team via a publicly available government data base [31].

##### Child-reported data: participation in school- and community-based active clubs

For all three waves, children completed a questionnaire and reported which days (Monday to Friday) they attended a sport or exercise after-school club based at their school; hereafter referred to as school-based active clubs. This was summed to give the number of days on which they attended a school-based active club (frequency) and a binary indicator (participation) of whether they attended at least one. Community-based active club participation was measured through the frequency in which they reported attending a sport or exercise club outside of school (0 = ‘Never’, 1 = ’1–2 days a week’, 2 = ‘3–4 days a week’, 3 = ‘5 + days a week’). These were coded with the midpoint number of days for each category to estimate the number of days on which they attended a community-based active club and a binary participation indicator. We also created total frequency and participation variables for attending either type of active club by adding the school-based and community-based active club frequencies.

##### Parent/carer-reported data: expenditure on community-based active clubs

In Waves 1 and 2, parent/carers reported the total weekly expenditure in UK pounds (£) on all community-based active clubs that their child was currently attending. Parents were also asked whether their child would have participated in more sport or exercise clubs external to school (community-based active clubs) if they were cheaper (yes/no).

##### School-reported data: provision of school-based active clubs

In Waves 1 and 2, schools reported school-based club provision for Year 6 pupils in the current school term, including the type of club, cost to parents and average number of Year 6 children attending. Clubs were classified as either active (e.g., sports, dance, outdoor play) or not active (e.g., art, cooking, coding). The cost charged to parents was reported either per session or per term and recoded to give the estimated cost for each after-school club and a binary indicator of whether the club was free to parents. Schools were asked how the school funded each club, collected as free text. This was cleaned and classified based on the responses received as paid for by parents, partially/wholly subsidised to parents (e.g., by PE and Sports Premium), run by school staff/volunteers, externally provided, or not known/missing.

#### Statistical analysis

Child characteristics and missing data were summarised by wave. All statistical analysis was conducted in Stata version 17 [32]. The analysis falls into three parts:

##### Child participation in active clubs

The percentage of children who participated in school-based active clubs, community-based active clubs and total active clubs was reported by gender and household education for each wave. We also reported the frequency of attendance (days per week) for those who participated in each type of club. For Waves 1 and 2, we additionally reported active club participation and frequency by FESS quartile. Child participation for each type of active club was modelled using logistic mixed effects regression, with children nested within schools and an independent variable for wave to capture differences between waves. Models were adjusted for child age, gender and household education. We reported odds ratios (ORs) for participation in the respective active clubs at Waves 1 and 2, compared to Wave 0. In addition, we modelled the frequency of attending each type of active club using Poisson mixed effects models, which are suitable for modelling count data such as the number of days of club attendance and account for child and school-level clustering. Models included a wave indicator and were adjusted for child age, gender and household education as before. We reported rate ratios (RRs) for Waves 1 and 2, which are multiplicative differences in the number of days attending the club(s) compared to Wave 0.

##### Parent/carer-reported expenditure

Total weekly parental expenditure on community-based active clubs was summarised by wave, and by gender, household education and FESS quartile. Costs were calculated only for those children who reported attending community-based active clubs. We also reported the estimated mean and median weekly expenditure per club between Waves 1 and 2. All cost data are presented in UK pounds (£).

##### School-level provision of active clubs

We reported descriptive summaries only, as there were only 23 and 27 schools in 2021 and 2022 respectively. We reported the average number of clubs provided and number of Year 6 children attending for all clubs and active clubs per school. We also reported cost data on the average cost of an active club to parents, and the source of funding.

### Qualitative methods

#### Participants and procedure

Parents and children who had participated in Waves 1 or 2, and school staff were invited to participate in a follow-up qualitative study. All participants had consented to being recontacted. One-to-one semi-structured interviews were conducted with parents and school staff and focus groups conducted with children. As recompense for their time, parents and school staff were provided with a £10 gift voucher.

Data were collected between August and December 2021 in Wave 1 and between February and July 2022 in Wave 2. Equal ratios of children with low/medium/high activity were drawn from each school (based on accelerometer-measured MVPA; see main study for further details of accelerometer protocols and collection [12, 15]). Index of Multiple deprivation (IMD) [29] was determined from parent and school postcodes, and was reported in deciles, with lower deciles indicating a greater level of deprivation.

#### Qualitative study materials

Topic guides were developed for each participant group (parents, school staff, and children) in both waves. Wave 1 topic guides focused on changes to physical activity and screen-viewing behaviour during periods of lockdowns and school closures (March 2020 – March 2021) and the short-term recovery period following this (April 2021 – December 2021), and factors that may have influenced these changes. Wave 2 topic guides focused on exploring these changes from a medium-term perspective (January - July 2022). All topic guides are provided in Supplementary File 2.

#### Qualitative data analysis

The framework method was used to support qualitative data analysis [33]. This method was selected to help generate themes by identifying commonalties and relationships between data. This method consisted of seven stages: (1) verbatim transcribing of interview/focus group audio recordings by a university-approved transcription service; (2) data familiarisation; (3) coding; (4) developing a working analytical framework; (5) applying the analytical framework; (6) charting data into the framework matrix; and (7) interpreting the data. In both waves, two transcripts for each participant group were independently coded and a separate codebook collaboratively developed for each participant group that was then applied to the remaining transcripts. Qualitative data was managed using NVivo version 12 [34]. Further information related to researcher reflexivity and qualitative analysis can be seen in Supplementary File 2.

## Results

### Quantitative results

Child and parent age and gender were similar between waves, but more participants were from households with higher educational qualifications in Waves 1 and 2 (Table 1). Schools taking part in each wave were similar in terms of urban/rural, neighbourhood deprivation and percentage of children receiving free school meals (Table 1). Missing data are reported in Supplementary Table 1.

**Table 1:** Characteristics of quantitative study participants by wave

	Wave 0 Pre-COVID-19 Mar 2017-May 2018 N = 1296		Wave 1 Jun 2021-Dec 2021 N = 393		Wave 2 Jan 2022-Jul 2022 N = 463	
**CHILD**						
**Age: mean (SD)**	11.0	(0.4)	10.9	(0.4)	11.1	(0.3)
**Gender: N (%)**						
Male	616	(48%)	198	(50%)	212	(49%)
Female	680	(52%)	183	(49%)	224	(51%)
**PARENT**						
**Parent age: N (%)**						
<39 yrs	248	(23%)	118	(30%)	121	(28%)
40–44 yrs	414	(39%)	136	(35%)	147	(34%)
45 + yrs	401	(38%)	134	(35%)	161	(38%)
**Parent gender: N (%)**						
Male	294	(27%)	91	(23%)	97	(23%)
Female	794	(73%)	297	(77%)	332	(77%)
**Household education: N (%)**						
Up to A level/equivalent	555	(47%)	131	(34%)	162	(38%)
Degree/ equivalent or higher	636	(54%)	257	(66%)	267	(62%)
**SCHOOL**	N = 50		N = 23		N = 27	
Urban: N (%)	45	(90%)	19	(83%)	24	(89%)
school IMD: mean (SD)	16.7	(15.3)	13.7	(10.2)	14.0	(10.9)
% free school meals: mean (SD)	10.2	(8.5)	12.9	(7.7)	12.9	(8.4)

A level = qualification at age 18. SD = standard deviation. IMD= Index of Multiple Deprivation; higher scores indicate more deprived areas

#### Child participation in active clubs

Participation in total active clubs was similar across waves (Table 2). However, the percentage of children who participated in a school-based active club was higher post-lockdown, rising from 43% in Wave 0 to 50% in Wave 2, while those who participated in a community-based active club was lower, dropping from 80 to 74%. Participation in school-based clubs was similar by gender, but girls were less likely to participate in community-based active clubs at all waves, with 72% in Wave 2 compared to 77% of boys. Children from households with lower educational qualifications were less likely to participate in both types of active club, especially community-based active clubs where 61% of children from lower education households attended compared to 82% of children from higher education households. Modelled difference in child participation in active clubs, adjusted for age, gender and household education found that children were less likely to participate in community-based active clubs post-lockdown (Wave 1 OR = 0.66, 95% CI: 0.49–0.88; Wave 2 OR = 0.65, 95% CI: 0.49–0.87) compared to pre-COVID-19 (Supplementary Table 2).

**Table 2:** Participation in community-based, school-based, and any active club by wave

	School-based active clubs			Community-based active clubs			Total active clubs		
	Wave 0	Wave 1	Wave 2	Wave 0	Wave 1	Wave 2	Wave 0	Wave 1	Wave 2
**Total**	43%	46%	50%	80%	75%	74%	84%	83%	85%
**Gender**									
Male	43%	49%	50%	82%	79%	77%	85%	86%	86%
Female	43%	44%	50%	77%	72%	72%	84%	82%	83%
**Household education**									
Up to A-level	38%	42%	47%	75%	67%	61%	80%	74%	76%
Degree or higher	48%	48%	52%	85%	79%	82%	88%	88%	89%
**Financial strain (FESS)**									
Q1 (lower strain)	-	56%	48%	-	81%	80%	-	93%	88%
Q2	-	45%	52%	-	82%	79%	-	86%	91%
Q3	-	43%	51%	-	74%	74%	-	82%	83%
Q4 (higher strain)	-	38%	48%	-	62%	67%	-	70%	78%

% represent proportions of children of each wave. Financial strain was not collected in Wave 0. FESS = family economic strain scale

On average, children participating in school-based active clubs attended 0.3 fewer clubs post-lockdown, with those attending on three or more days halving from 19% in Wave 0 to 10% in Wave 2, while those attending only one day increased from 56 to 73% (Supplementary Tables 3 and 4). Frequency was similar for boys and girls at all waves, but children from households with lower educational qualifications attended fewer clubs, with the observed post-lockdown drop mainly among those from higher educational households. The frequency of attending community-based active clubs was slightly lower post-lockdown, with the percentage of children attending on five or more days 15% and 9% at Waves 0 and 2 respectively. Girls and children from lower education households attended fewer community-based active clubs at all waves. Displayed in Supplementary Table 5, modelled attendance frequency for school-based active clubs, adjusting for age, gender and household education, was 19% lower in Wave 2 (RR = 0.85, 95% CI: 0.71–0.93), compared to pre-COVID-19, and attendance frequency for community-based active clubs was 8% lower (RR = 0.92, 95% CI: 0.85–1.01).

#### Parent/carer-reported expenditure

Table 3 presents parents’ total expenditure per week on community-based active clubs. Median weekly expenditure was £5 higher in Wave 2 than Wave 1 (£15 and £10 respectively), with a median of £6.67 per session in Wave 2. Weekly expenditure for girls was higher than for boys by £2.75 in Wave 1 and £3.00 in Wave 2. Expenditure was similar across household education levels for both waves, and similar across financial strain quartiles in Wave 1, but with lower expenditure in the highest quartile (most financial strain) in Wave 2. The percentage of parents who reported that their children would attend more community-based active clubs if they were cheaper rose from 38% in Wave 1 to 44% in Wave 2 overall, and increased from 73 to 76% among those reporting the most financial strain (Supplementary Table 6).

**Table 3:** Total weekly parental expenditure community-based active club at Waves 1 and 2

	Wave 1		Wave 2	
	Median (IQR)		Median (IQR)	
Total expenditure	£10.00	(£14.00)	£15.00	(£17.00)
Approx per session	£5.71	(£5.33)	£6.67	(£5.71)
**Gender**				
Male	£10.00	(£15.00)	£12.00	(£13.00)
Female	£12.75	(£18.50)	£15.00	(£15.50)
**Financial strain**				
Q1 (less strain)	£12.00	(£14.00)	£15.00	(£15.00)
Q2	£12.00	(£14.00)	£15.00	(£18.00)
Q3	£10.00	(£9.50)	£15.00	(£14.00)
Q4 (more strain)	£11.50	(£14.00)	£10.00	(£23.00)
**Household education**				
Up to A level	£10.00	(£13.50)	£15.00	(£18.00)
Degree or higher	£11.50	(£14.00)	£15.00	(£17.00)

Expenditure reported for those who use clubs. IQR = interquartile range

#### School-level provision of active clubs

The distribution of Year 6 clubs offered by schools changed between Waves 1 and 2 (Supplementary Table 7), with the average number of all clubs per school increasing from 3.8 to 5.0. Of these, over two thirds were active clubs. The average number of active clubs per school increased from 2.6 in Wave 1 to 3.4 in Wave 2, with the percentage of schools offering 5 or more active clubs per week increasing from 13 to 27%.

A large proportion of active clubs were provided free to parents (42% in Wave 1 and 50% in Wave 2), with paid active clubs costing parents a median of £3.25 per session in Wave 1 and £3.88 in Wave 2 (Supplementary Table 7). In Wave 1, 27% of school-based active clubs were partially/wholly subsidised to parents, and 27% were run voluntarily by school staff or other volunteers, increasing to 34% and 33% in Wave 2 respectively. Only 5–10% of school-based active clubs were funded externally (Supplementary Table 7).

### Qualitative results

In total, 43 parent interviews (ranging from 27 to 75 min), 12 child focus groups with 92 children from 12 schools (ranging from 33 to 61 min), and 18 school staff interviews from 12 different schools (ranging from 33 to 59 min) were conducted. Table 4 displays participant demographic information.

**Table 4:** Characteristics of parents and school staff interviewees and child focus group participants by wave

	Wave 1	Wave 2	Total
***Parents***	***21***	***22***	***43***
**Gender**			
Male	0	7	7
Female	21	15	36
**Parent activity levels**			
High MVPA	11	12	23
Medium MVPA	9	5	14
Low MVPA	1	5	6
Insufficient data	0	1	1
**IMD decile**			
≤ 5 (higher deprivation)	4	5	9
> 5 (lower deprivation)	17	17	34
**Parent education**			
Higher degree	9	4	13
Degree	7	16	23
A level	5	2	7
***Children***	***47***	***45***	***92***
**Gender**			
Male	26	22	48
Female	21	23	44
**Child activity levels**			
High MVPA	16	11	27
Medium MVPA	16	17	33
Low MVPA	15	17	32
**IMD decile**			
≤ 5 (higher deprivation)	17	15	32
> 5 (lower deprivation)	30	30	60
***School staff***	***9***	***9***	**18**
**Gender**			
Male	3	5	8
Female	6	4	10
**Role**			
Year 6 teacher	7	5	12
Full-time PE Coordinator	1	2	3
Deputy/ Headteacher	1	2	3

3 parents and 5 school staff participated in both waves. MVPA = moderate-vigorous physical activity. IMD - Index of Multiple Deprivation

#### Qualitative analysis

Three themes were generated related to children’s active club participation over the year following the COVID-19 lockdowns in England. These were: (1) Community-based active clubs as a luxury post-COVID-19 restrictions; (2) Negative impact of the pandemic on the community-based active club environment; and (3) Many schools struggled to meet increased demand for clubs. Themes were constructed around a central organising concept and all themes were reflected within data across all participant groups.

##### Theme 1: community-based active clubs as a luxury post-COVID restrictions

This theme explores the impact of financial pressures on children’s community-based active club participation. Parents described the impact of the cost-of-living crisis on their families and worries about the future UK economy were evident, leading many parents to be more cautious with their spending. Subsequently, community-based active clubs had become a luxury many felt they could not afford. A sense that community-based clubs were designed for middle-class families with disposable income was evoked by some parents, suggesting inequalities between socioeconomic groups.

“*…I wouldn’t think about signing up for a new class or something because I would be definitely thinking about trying to just save money if we can… I anticipate rising costs of living will impact my family… if we needed to make cuts then it would be that we can’t afford to pay for [Child’s name] to do gym [gymnastics] or things like that...They’re really expensive, doing clubs out[side] of school… it’s [community-based active clubs] only for the people who have money.” (Wave 2, parent 13, IMD Decile 10)*

*“…it [community-based active club] got too expensive, so we had to quit.” (Wave 1, child focus group 2, unknown gender)*

Luxuries were not limited to the financial domain, but extended to parents’ available time, whereby increased working pressures and hours to offset lost income led to a sense of busy-ness in the workplace, suggesting that the demand for work may have increased. As a result of increased work pressures and hours, parents felt that their availability to provide the necessary logistical support for their child’s community-based active club participation, such as transport to and from the venue, was reduced.

*“…work has ramped up tenfold. You’re delivering a lot more than you ever were before.” (Wave 2, parent 18, IMD Decile 10)*

*“…people are having to work more because they need more money because everything is costing more money… So where I turn jobs down, there were people that took my job who have got kids. …” (Wave 2, parent 20, IMD Decile 10)*

##### Theme 2: negative impact of the pandemic on the community-based active club environment

This theme highlights the perceived negative impact of the pandemic on active club opportunities in the community. Participants spoke of challenges that some community-based active clubs experienced when re-opening/re-establishing themselves after the lifting of COVID-19 restrictions, with many describing reduced or changed opportunities that created feelings of disappointment for some children. A loss of interest among some children for the community-based active club they previously attended was also noted, reflecting the change in activity and sedentary habits formed over the pandemic explored in related research [14, 16].

*“[Child’s name] was doing swimming before COVID and then that never quite got started again...” (Wave 2, parent 21, IMD Decile 7)*

*“Before all the COVID and stuff, I used to go to tennis and football but now I just do gymnastics… I just lost interest during the lockdown of football and tennis.” (Wave 2, child focus group 1, girl)*

The reduction in children attending the more costly community-based active clubs was suggested to have negatively impacted the quality of the club environment. Participants felt that finding other teams to organise competitions with had become difficult following the pandemic, reducing what can be a key element of enjoyment for some children. Others described challenges in finding volunteers to help organise clubs and competitions, a product of parents having less available time (Theme 1).

*“…if the [community-based] clubs can’t get enough teams, they’re not going to be able to have competition… my youngest son, he’s starting football, so I’ve just set up an under 8s for him for this year. But in Bristol for a Saturday morning, they can only put in 3 teams out of the whole of Bristol. So these football teams aren’t being set up because there’s not the people to do it because they’re too busy doing other things…” (Wave 2, parent 20, IMD Decile 10)*

*“I used to play basketball but then after COVID…it got less fun because they couldn’t find as many people to play against...” (Wave 1, child focus group 1, boy)*

Participating in community-based active clubs to socialise with friends was also evoked. Children described the challenges they experienced when their friends did not return to their club and needing to connect with new, unfamiliar children. These difficulties may be exacerbated by social challenges that children experienced post-lockdown [13, 16].

*“…when we went back [to community-based active clubs], because I went back on my own, I didn’t know anyone… I have my friend now, but a lot of them [other children at the club] will just have their friend group and they didn’t really talk to me because I’m the youngest and the smallest there, so they just ignore me…” (Wave 2, child focus group 1, girl)*

##### Theme 3: many schools struggled to meet increased demand for clubs

This theme describes an increased demand for school-based active clubs that the over-pressured school environment was unable to meet. In the context of the financial and time challenges facing families (Theme 1), parents and school staff spoke of an increased demand for school-based active clubs among children. School-based active clubs were framed as a more affordable alternative to community-based active clubs, which can be offered at a reduced cost or free of charge and, when following on from the school day, do not require significant time investment from parents/carers for transportation.

*“…they’re reintroducing the after-school clubs. I’ve put [Child’s name] and [Sibling’s name] into the netball club… partly because it was free, because again I was spending so much money. Even swimming, it was so expensive… So I had to pick what I could afford or not afford… The netball thing is free and I’m like, ‘If it’s free, you can go for it.’” (Wave 2, parent 12, IMD decile 4)*

*“We both work… [our work] can go into evenings as well. So it becomes bound to the logistics of what can you actually get them to…. there isn’t much independence from a 10–11-year-old to, kind of, get themselves to certain things, you know, it’s a logistical nightmare, sometimes, ferrying children to and from these activities.” (Wave 2, parent 1, IMD Decile 8)*

Lockdowns and restrictions challenged an already over-pressured primary school, as school staff tried to meet the complex and varying post-lockdown needs of each pupil [17]. Subsequently, this has impacted the provision of school-based active clubs that can require a significant time commitment from school staff, usually on a voluntary basis [17]. Despite increased pressures, many school staff noted a greater provision of school-based active clubs in 2022, yet meeting the increased demand was challenging. Parents and school staff spoke of waiting lists and the speed with which school-based active clubs became fully booked. Unable to meet the increased demand for school-based active clubs, some schools alternated attendance among pupils to ensure that all have the opportunity to participate in at least one active club. However, this led to many remaining on a waiting list.

*“I wish there were more after-school clubs at [Child’s name]’s school. There are an awful lot of children in the school and they’ve only got so many spaces, particularly for sports clubs. So when it’s time to sign your child up for an after-school club, the places go very fast. They’re usually vastly oversubscribed…” (Wave 2, parent 3, IMD Decile 8)*.

*“Across the school we can offer, I don’t know, seven or eight clubs a week… just because we’ve got extra members of staff in… we’ve got higher uptake than we had prior to COVID. We do sometimes still have waiting lists… we had to say, “Some of you can do it this half term, the ones that didn’t get space this half term will have to do it next half term”. (Wave 2, school staff 3, dedicated PE coordinator)*

## Discussion

**Fig. 2:**
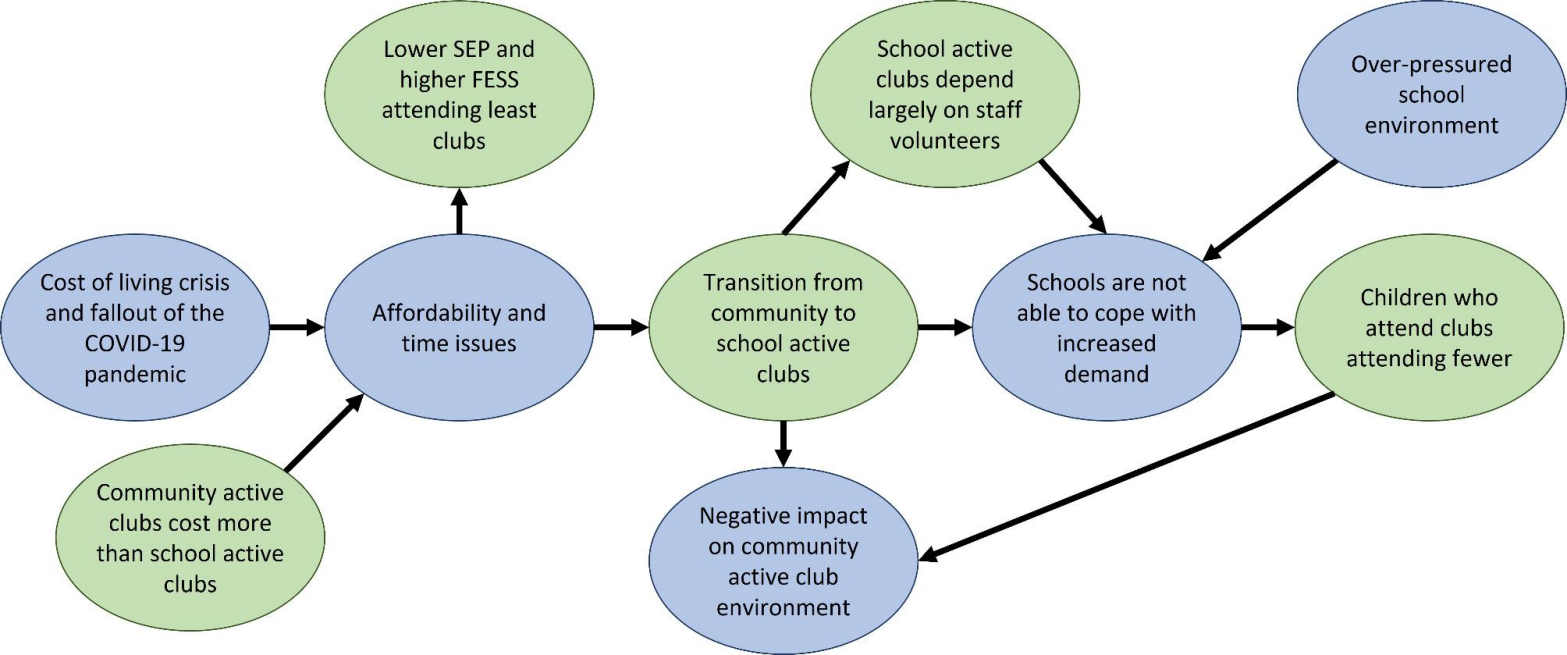
Mixed methods narrative of the impact of the cost-of-living crisis and COVID-19 pandemic on children’s active club participation Blue = qualitative findings; green = quantitative findings. SEP= Socioeconomic position

This study combined rich data from multiple sources to provide a novel insight into the current status of active club participation, provision, and costs. The overall study narrative is summarised in Fig. 2. Our results indicate that the fallout of the COVID-19 pandemic and cost-of-living crisis has created hardships for many families, leading to reduced disposable income and time to support children’s activities. School-based active clubs were cheaper, often subsidised or free, and parents found them more convenient than community-based active clubs which were seen as a luxury not all were able to afford. This was reflected in the quantitative data, with participation in school-based active clubs higher and participation in community-based active clubs lower post-lockdown, especially among children from families with lower education attainment and/or those experiencing greater financial strain. This shift has negatively impacted the community-based active club environment, as fewer children are able to attend and adults less available to volunteer. While school-based active club provision has increased between Waves 1 and 2, with a rise in subsidised places and volunteer-run clubs, increased pressures within the primary school environment [17] mean that many schools are struggling to meet this larger demand for school-based active clubs.

Meeting the increased demand for school-based active clubs offers a promising intervention area to help promote physical activity among children following COVID-19 that aligns with the new normal and its greater dependence on active clubs [13]. We found that while more children attended school-based active clubs overall, they attended fewer sessions per pupil, as schools tried to provide opportunities to all children within the over-pressured school environment that relied largely on school staff/volunteers [17]. The PE and Sports premium funding [25], funding from the English government for primary schools to promote physical activity, was a key enabler for schools to provide active clubs, with a third of school-based active clubs partially/wholly subsidised to parents through this. The importance of government funding has been echoed in third sector reports that claimed 68% of schools reported that they would be unable to continue after-school sport sessions without the PE and Sports Premium [35]. In a time of economic crisis, a reduction in low-cost and convenient school-based active clubs could have a substantial negative impact on children’s physical activity, which has been found to be sensitive to post-COVID-19 disruptions in physical activity provision [15]. The targeting of existing local and national funding to support school-based active clubs could therefore be a key practical option for increasing children’s physical activity at a population level.

Cost and location were frequently reported as barriers to sport participation pre-pandemic [36]. In this study, community-based active clubs, which we observed cost a median of £6.67 per session and required significant amount of parent’s time, were viewed as a luxury that many were not able to afford. Subsequently, children from families experiencing higher financial strain and/or lower educational attainment, an indicator of socioeconomic position, have been most impacted. Prior to the pandemic, there was mixed evidence for socioeconomic differences in overall physical activity among children [37–39], though household socioeconomic position was related to sport participation [40]. The data reported here suggest that socioeconomic issues may have widened as a result of the COVID-19 pandemic and cost-of-living crisis. We also observed that parental expenditure for girls’ community-based active clubs was higher than for boys and participation among girls was lower. Gender inequalities in children’s physical activity existed prior to the pandemic, with girls participating in less physical activity on average than boys [20, 37]. These disparities have continued following the return to school after the COVID-19 lockdowns [12, 41] and increased costs among girls may have become a more challenging issue in the context of the cost-of-living crisis. Strategies to address socioeconomic and gender disparities in active club participation are crucial to help these children access the new normal for children’s physical activity and prevent widening health inequalities.

Our results highlight the challenges faced by community-based active clubs and the impact of changes/reductions in children attending. Friendships and social networks play an important role in children and young peoples’ physical activity [42–46], particularly structured physical activity, such as active clubs [47]. The impact of a child’s friend no longer participating in the same active club may be particularly influential following the COVID-19 lockdowns where socialising with unfamiliar children may be more challenging [13, 16]. These social challenges may be a contributing factor towards the shift to school-based active clubs where children are able to participate with familiar children. This shift appears to be cyclical, whereby fewer children are attending community-based active clubs which reduces their quality which in turn encourages more children to leave the club. Interventions that draw upon social networks to promote physical activity in children and young people may therefore present an important area of research post-pandemic. However, to date, such interventions have had limited effect on physical activity behaviour among mixed gender [48–50] and female children and young people [51]. Reflecting upon the reasons why these interventions may not have shown an effect, researchers highlighted the importance of personal approaches to training social influence agents and ensuring they had high enough status to influence peers [49], focusing on a single behaviour (e.g. physical activity) [50], and making sure content was age-appropriate and training delivered outside of school [51]. It is important that we consider these lessons in the design of future interventions.

While current government and third sector organisation support in the UK for community-based active clubs [52–54] is valuable and timely, our findings imply that many may still be struggling to remain open and deliver quality opportunities with reduced numbers of children. It is therefore important that policymakers and practitioners continue this support to increase affordable and convenient community-based opportunities and encourage greater numbers of children to participate, especially girls and those from lower socioeconomic backgrounds. Community-based active clubs may benefit from working with schools to provide onsite, low cost opportunities that may subsequently ease some of the pressures facing schools.

### Key findings and implications

The overall finding of this study is a shift from community-based active clubs to school-based active clubs, driven by the challenges of the cost-of-living crisis and fallout of the COVID-19 pandemic, with community clubs struggling to remain open while many schools are not able to meet the increased demand. As a result, many children were not participating in the new physical activity normal and socioeconomic and gender disparities were evident. Thus, the implications of this study are:

1. Support needs to be provided to state primary schools to help them increase provision through targeted use of existing funding to meet the greater demands for school-based active clubs.
2. Provision of affordable and convenient community-based active clubs needs to be developed and expanded.
3. National and local governments need to support existing community-based active clubs, especially those who are at risk of closing due to insufficient numbers of children.
4. Targeted funding and measures to prevent socioeconomic and gender disparities in active club participation are needed to reduce current and future health inequalities.
5. Implementation of strategies to prevent declines in community-based active club participation, especially among girls, through positive social interactions.

### Strengths and limitations

A key strength of this study is the mixed-methods design, which drew upon a unique combination of data collected from schools, parents, and children at three separate timepoints. This allowed a multi-perspective exploration of issues that provided nuanced findings. However, our understanding of community-based active club provision and the providers’ perspectives were limited as this was not an objective of the Active-6 project. As a result, we relied on perceptions of children who participated and their parents, rather than the organisations/organisers themselves, and so our findings related to the negative impact on community-based active clubs warrant further research that explores issues from the providers’ perspective. Furthermore, cost data were collected from two different sources and may be subject to measurement error that make direct comparisons challenging. We were also unable to separate issues stemming from the COVID-19 pandemic and the cost-of-living crisis, which may limit our understanding of the context that led to issues identified in this study. Finally, nonresponse bias, may result in differences in the sample between waves, although we have included sociodemographic characteristics in our model which will reduce this to some extent. Further research is warranted to explore the school environment in more detail and future studies may benefit from considering these issues in their design.

## Conclusions

This study provides insight into the current status of active club participation, provision and costs. We found a shift from community-based to school-based active clubs, driven by the cost-of-living crisis and fallout of the COVID-19 pandemic. This negatively impacted the community-based active club environment and may be discouraging children from participating. However, primary schools were not always able to meet the increased demand for school-based active clubs. We also identified potential socioeconomic and gender disparities. It is therefore vital that policymakers provide support to schools to help them meet increased demand for active clubs, increase quality provision of community-based active clubs and provide additional support to those who are at risk of closing, and target funding to address socioeconomic and gender inequalities.

### Electronic supplementary material

Below is the link to the electronic supplementary material.

Supplementary Material 1: Additional tables.

Supplementary Material 2: Further qualitative study information.

## Data Availability

Data are available on application at the University of Bristol data repository, https://data.bris.ac.uk, at 10.5523/bris.32kncke12keg22v9tr5etxah0d.

## Abbreviations

MVPA: Moderate to Vigorous Physical Activity
COVID-19: Coronavirus Disease 2019
FESS: Family Economic Strain Scale
IMD: Index of Multiple Deprivation
OR: Odds Ratio
RR: Rate Ratio
