# Supplementary material for: The status of active after-school clubs among primary school children in England (UK) after the COVD-19 lockdowns: implications for policy and practice
Source: Int J Behav Nutr Phys Act. 2023 Oct 5;20:120. doi: 10.1186/s12966-023-01499-x (PMC10552244; doi:10.1186/s12966-023-01499-x)
Supplement: Supplementary file 1 — Supplementary Material 1: Additional tables. [file 12966_2023_1499_MOESM1_ESM.docx]

**Supplementary file 1: Additional tables**

**The status of active after-school clubs among primary school children in the UK after the COVD-19 lockdowns: Implications for policy and practice.**

Robert Walker, Ruth Salway, Danielle House, Lydia Emm-Collison, Katie Breheny, Kate Sansum_,_ Sarah Churchward, Joanna G Williams, Frank de Vocht, William Hollingworth, and Russell Jago

**Additional Tables**

Supplementary Table 1: Missing data

Supplementary Table 2: Odds ratios for differences in club participation by wave

Supplementary Table 3: Frequency (average number of days) in community-based, school-based, and total active club among children who participated in at least one of those clubs by wave

Supplementary Table 4: Distribution of days attending community-based and school-based active clubs among children who participated in those clubs by wave

Supplementary Table 5: Rate ratios (RRs) for differences in frequency of club attendance by wave, amongst those participating

Supplementary Table 6: Percentage of parents who responded their child would have participated in more community-based active clubs if they were cheaper

Supplementary Table 7: School provision of extracurricular clubs, number of Year 6 children attending, cost to parents, and sources of funding by wave

**Supplementary Table 1: Missing data**

|  | Wave 0  Mar 2017-May 2018  N=1296 | | Wave 1  Jun 2021-Dec 2021  N=393 | | Wave 2  Jan 2022-Jul 2022  N=463 | |
| --- | --- | --- | --- | --- | --- | --- |
|  | Missing | (%) | Missing | (%) | Missing | (%) |
| **Socio-demographics** |  |  |  |  |  |  |
| Child age | 0 | (0%) | 0 | (0%) | 0 | (0%) |
| Child gender | 0 | (0%) | 0 | (0%) | 0 | (0%) |
| Household education | 105 | (8%) | 5 | (1%) | 7 | (2%) |
| Family financial strain^1^ | - |  | 33 | (8%) | 34 | (8%) |
| **Child participation** |  |  |  |  |  |  |
| School-based active clubs | 11 | (1%) | 24 | (6%) | 27 | (6%) |
| Community-based active clubs | 2 | (<1%) | 24 | (6%) | 28 | (6%) |
| Total active clubs | 11 | (1%) | 24 | (6%) | 28 | (6%) |
| **Parental expenditure** |  |  |  |  |  |  |
| Parental expenditure^1^ | - |  | 42 | (11%) | 50 | (11%) |
| Would club use if cheaper^1^ | - |  | 35 | (9%) | 37 | (8%) |
| **School clubs**^1^ |  |  | N=87 |  | N=131 |  |
| Type of club | - |  | 0 | (0%) | 0 | (0%) |
| No. Y6 children | - |  | 22 | (25%) | 24 | (18%) |
| Cost to parents | - |  | 13 | (15%) | 19 | (15%) |
| Funding source | - |  | 15 | (17%) | 25 | (19%) |

^1^ not collected in Wave 0

**Supplementary Table 2: Odds ratios for differences in club participation by wave**

|  | Wave 1 | | Wave 2 | |  |
| --- | --- | --- | --- | --- | --- |
|  | OR^1^ | 95% CI | OR^1^ | 95% CI | p-value^2^ |
| School-based active clubs | 1.00 | (0.77, 1.30) | 1.19 | (0.93, 1.53) | 0.370 |
| Community-based active clubs | 0.66 | (0.49, 0.88) | 0.65 | (0.49, 0.87) | 0.002 |
| Total active clubs | 0.80 | (0.57, 1.11) | 0.90 | (0.65, 1.25) | 0.390 |

^1^ OR compared to Wave 0

^2^ P-value for test of no difference between waves

All models adjusted for child age, gender and household education. OR=odds ratio. CI=confidence interval

**Supplementary Table 3: Frequency (average number of days) in community-based, school-based, and total active club among children who participated in at least one of those clubs by wave**

|  | School-based active clubs | | | | | | Community-based active clubs | | | | | | Total active clubs | | | | | |
| --- | --- | --- | --- | --- | --- | --- | --- | --- | --- | --- | --- | --- | --- | --- | --- | --- | --- | --- |
|  | Wave 0 | | Wave 1 | | Wave 2 | | Wave 0 | | Wave 1 | | Wave 2 | | Wave 0 | | Wave 1 | | Wave 2 | |
| Total | 1.7 | (1.0) | 1.6 | (0.9) | 1.4 | (0.8) | 2.6 | (1.3) | 2.3 | (1.2) | 2.5 | (1.2) | 3.3 | (1.9) | 3.0 | (1.7) | 3.0 | (1.6) |
| **Gender** |  | |  | |  | |  | |  | |  | |  | |  | |  | |
| Male | 1.7 | (1.0) | 1.6 | (0.8) | 1.4 | (0.7) | 2.8 | (1.4) | 2.4 | (1.1) | 2.6 | (1.3) | 3.6 | (1.9) | 3.1 | (1.7) | 3.2 | (1.6) |
| Female | 1.7 | (1.0) | 1.7 | (0.9) | 1.4 | (0.9) | 2.4 | (1.3) | 2.2 | (1.2) | 2.3 | (1.2) | 3.1 | (1.9) | 2.9 | (1.7) | 2.8 | (1.6) |
| **Household education** |  | |  | |  | |  | |  | |  | |  | |  | |  | |
| Up to A-level | 1.5 | (0.9) | 1.6 | (1.0) | 1.5 | (1.0) | 2.6 | (1.3) | 2.3 | (1.1) | 2.3 | (1.2) | 3.1 | (1.8) | 2.0 | (1.7) | 2.7 | (1.6) |
| Degree or higher | 1.9 | (1.1) | 1.6 | (0.8) | 1.4 | (0.7) | 2.7 | (1.4) | 2.3 | (1.2) | 2.5 | (1.2) | 3.6 | (2.0) | 2.9 | (1.7) | 3.1 | (1.6) |
| **Financial strain** (FESS) |  | |  | |  | |  | |  | |  | |  | |  | |  | |
| Q1 (lower strain) | - | | 1.6 | (0.8) | 1.4 | (0.7) | - | | 2.5 | (1.2) | 2.8 | (1.3) | - | | 3.2 | (1.8) | 3.3 | (1.6) |
| Q2 | - | | 1.7 | (0.9) | 1.4 | (0.9) | - | | 2.2 | (1.1) | 2.4 | (1.3) | - | | 2.9 | (1.7) | 2.9 | (1.6) |
| Q3 | - | | 1.6 | (1.0) | 1.3 | (0.8) | - | | 2.1 | (1.0) | 2.2 | (1.1) | - | | 2.8 | (1.5) | 2.8 | (1.6) |
| Q4 (higher strain) | - | | 1.5 | (0.6) | 1.5 | (0.8) | - | | 2.3 | (1.1) | 2.4 | (1.1) | - | | 2.8 | (1.4) | 3.0 | (1.6) |

Note: financial strain was not collected in Wave 0

**Supplementary Table 4: Distribution of days attending community-based and school-based active clubs among children who participated in those clubs by wave**

|  | | Wave 0 | | Wave 1 | | Wave 2 | |
| --- | --- | --- | --- | --- | --- | --- | --- |
|  | | N | % | N | % | N | % |
| School-based active clubs | |  |  |  |  |  |  |
|  | 1 day | 310 | 56% | 99 | 58% | 147 | 73% |
|  | 2 days | 139 | 25% | 47 | 28% | 34 | 17% |
|  | 3+ days | 104 | 19% | 24 | 14% | 21 | 10% |
| Community-based active clubs | |  |  |  |  |  |  |
|  | 1-2 days | 570 | 56% | 179 | 64% | 179 | 59% |
|  | 3-4 days | 299 | 29% | 79 | 28% | 97 | 32% |
|  | 5 days | 153 | 15% | 20 | 7% | 28 | 9% |

**Supplementary Table 5: Rate ratios (RRs) for differences in frequency of club attendance by wave, amongst those participating**

|  | Wave 1 | | Wave 2 | |  |
| --- | --- | --- | --- | --- | --- |
|  | RR^1^ | 95% CI | RR^1^ | 95% CI | p-value^2^ |
| School-based active clubs | 0.92 | (0.80, 1.06) | 0.81 | (0.71, 0.93) | 0.011 |
| Community-based active clubs | 0.86 | (0.79, 0.94) | 0.92 | (0.85, 1.01) | 0.002 |
| Total active clubs | 0.84 | (0.78, 0.91) | 0.85 | (0.79, 0.92) | <0.001 |

^1^ RR compared to Wave 0

^2^ P-value for test of no difference between waves

All models adjusted for child age, gender and household education. RR=rate ratio. CI=confidence interval.

**Supplementary Table 6: Percentage of parents who responded their child would have participated in more community-based active clubs if they were cheaper**

|  | Wave 1 | Wave2 |
| --- | --- | --- |
| Total | 38% | 44% |
| **Gender** |  |  |
| Male | 35% | 42% |
| Female | 41% | 46% |
| **Household education** |  |  |
| Up to A-level | 48% | 52% |
| Degree or higher | 32% | 40% |
| **Financial strain (FESS)** |  |  |
| Q1 (lower strain) | 16% | 24% |
| Q2 | 26% | 29% |
| Q3 | 42% | 46% |
| Q4 (higher strain) | 73% | 76% |

**Supplementary Table 7: School provision of extracurricular clubs, number of Year 6 children attending, cost to parents, and sources of funding by wave**

|  | Wave 1  (23 schools) | | | | Wave 2  (26 schools) | | | |
| --- | --- | --- | --- | --- | --- | --- | --- | --- |
|  | Clubs | | Schools | | Clubs | | Schools | |
| **Number of clubs** |  | |  | |  | |  | |
| All after school clubs | 87 | | 3.8 | | 131 | | 5.0 | |
| Active clubs | 60 | (68%) |  | | 88 | (69%) |  | |
| None |  |  | 1 | 4% |  |  | 1 | 4% |
| 1-2 clubs |  |  | 12 | 52% |  |  | 10 | 38% |
| 3-4 clubs |  |  | 7 | 30% |  |  | 8 | 31% |
| 5+ clubs |  |  | 3 | 13% |  |  | 7 | 27% |
| Mean |  |  | 2.6 | |  |  | 3.4 | |
| **Y6 children attending** |  | |  | |  | |  | |
| All after school clubs | 517 | | 22.5 | | 833 | | 32.0 | |
| Active clubs | 412 | (80%) | 17.9 | | 684 | (82%) | 26.3 | |
| **Costs to parents** |  | | | |  | | | |
| Free to parents: N (%) | 22 | (42%) |  | | 40 | (50%) |  | |
| Cost per paid club:  Median (IQR) | £3.25 | (£2.00) |  | | £3.88 | (£3.00) |  | |
| **Source of funding N(%)** |  | | | |  | | | |
| Parents only | 17 | (33%) |  | | 22 | (27%) |  | |
| Partially/wholly subsidised | 15 | (29%) |  | | 28 | (34%) |  | |
| School staff/volunteers | 14 | (27%) |  | | 27 | (33%) |  | |
| Externally provided | 5 | (10%) |  | | 4 | (5%) |  | |

IQR=interquartile range
