# Supplementary material for: The status of active after-school clubs among primary school children in England (UK) after the COVD-19 lockdowns: implications for policy and practice
Source: Int J Behav Nutr Phys Act. 2023 Oct 5;20:120. doi: 10.1186/s12966-023-01499-x (PMC10552244; doi:10.1186/s12966-023-01499-x)
Supplement: Supplementary file 2 — Supplementary Material 2: Further qualitative study information. [file 12966_2023_1499_MOESM2_ESM.docx]

**Supplementary file 2: Qualitative research information and topic guides**

**The status of active after-school clubs among primary school children in the UK after the COVD-19 lockdowns: Implications for policy and practice.**

Robert Walker, Ruth Salway, Danielle House, Lydia Emm-Collison, Katie Breheny, Kate Sansum_,_ Sarah Churchward, Joanna G Williams, Frank de Vocht, William Hollingworth, and Russell Jago

**Qualitative research information**

**Research team and reflexivity**

Robert Walker PhD is an experienced male qualitative researcher who was working as a Research Associate at the University of Bristol. He has designed and led multiple qualitative studies related to physical activity behaviour in diverse populations, including military veterans. Robert led data collection and analysis.

Byron Tibbitts MSc is a male who was working as a Senior Research Associate (Project Manager). He is an experienced qualitative researcher in relation to children’s physical activity. Byron left the University of Bristol in December 2021.

Danielle House PhD is an experienced female qualitative researcher with a diverse research background. She was working as a Senior Research Associate (Project Manager) at the University of Bristol at the time of data collection and analysis. Danielle joined the research team in December 2021.

Kate Sansum MSc is a female who was working as a fieldworker on the Active-6 project at the University of Bristol. She has an undergraduate and postgraduate degree in Sport and Exercise Science. Kate left the University of Bristol in July 2022.

Tom Reid BSc is an experienced male fieldworker who was working on the Active-6 project at the University of Bristol. He has an undergraduate degree in Sport and Exercise Science.

**Study design**

Topic guides for interviews and focus groups were collaboratively developed with the research team, which were then reflected on and adapted throughout the data collection process. Parent and school staff interview guides were all shared with participants in advance; however, this was not done for children as we did not think it was appropriate for this age group.

Wave 1 parent interviews were conducted between August and December 2021 by either RW, TR, or BT, whereas Wave 2 were conducted by RW between February and July 2022. All parent interviews were conducted remotely by telephone or video conference call. Only one researcher was present in the interviews.

Wave 1 school staff interviews were entirely remote and conducted by RW between November and December 2021, whilst Wave 2 consisted of one in-person and 8 remote interviews conducted by RW between May and July 2022. Only one researcher was present in the interviews.

Wave 1 child focus groups were facilitated by RW, TR, BT, and DH in December 2021, while Wave 2 focus groups were facilitated by RW, KS, and DH between May and June 2022. All focus groups took place at the children’s schools. RW facilitated all focus groups whilst TR, BT, KS, and DH made field notes and supported facilitation.

Researchers independently coded two transcripts from each participant group (Wave 1: RW, TR, BT, KS, DH; Wave 2: RW, KS, DH). Meetings were then held to discuss interpretations that helped promote reflexivity.

**Analysis and findings**

Participants were informed about the aims of the overall research project and specific aims of qualitative research through discussions at the beginning of interviews/focus groups and detailed participant information sheets. Participants knew the researchers’ gender and each briefly introduced themselves and their positionality at the beginning of each interview/focus group. None of the researchers held a relationship with any participants. All participants were given the opportunity to read transcripts once audio recordings were transcribed, but none requested to do so.

**Topic Guides**

**Wave 1 – August to December 2021**

1. **Parent interview guide**

**Introduction and consent**

Thank you for agreeing to take part in this interview, your views and opinions are really important to us.

In the interview today, I would like to talk about three main points:

1. You and your child’s physical activity patterns and how these may have been impacted by COVID-19.
2. Factors that you feel you have caused any changes
3. Whether you think there is anything that can be done to help support you and your child’s physical activity

We are really interested in your honest opinions, we are not here to judge you, and we do not want you to feel like you should answer any of the questions in a certain way. There are no right or wrong answers, and as much detail you can give on the topics as possible is really appreciated.

Before we get started, I’d like to go over some important information regarding the interview and your data:

- I will be **recording the conversation** to help us remember what you said
- You can ask for the recording to be stopped at any time
- After we have written a report about all the opinions we have heard from the parents taking part, the recordings will be destroyed
- We will also change any names or identifying information so none of the information that is written down and recorded can be connected to you in any way
- Please remember that you can interrupt the interview at any point if you need to.
- If you do not want to answer a question please say so.
- Are you comfortable to proceed with the interview?

*If the parent says yes the recording will start. As noted on the information sheet the interview will be recorded.*

- For the recording, can you please confirm that you have been given information explaining about the study and that you understand what this project is about?
- Can you please confirm that you have had the opportunity to ask questions and discuss this study?
- Can you please confirm that you are aware that the interview data will be stored anonymously and securely for 20 years?
- Can you please confirm that you are aware that you are free to withdraw your data from the study at any time up to three weeks after this interview has taken place **[X DATE]**, and that you do not need to give a reason for withdrawing?
- And finally, can you please confirm that you are happy to take part in the interview?

**Icebreakers**

1. Discussion of physical activity definition (4 domains)
   1. Leisure-time
   2. Transport
   3. Household
   4. Occupational
2. Can you tell us what your child in Year 6’s favourite physical activity/physically active thing to do is and why do you think this is their favourite?
3. Can you describe your favourite physical activity/physically active thing to do is and why do you think this is their favourite?

**Changes in PA and device/screen time over COVID pandemic**

***Pre-lockdown***

1. How would you describe your activity levels before the first lockdown?
   - Did you often use active modes of transport?
   - Were you part of any active clubs or have any active hobbies?
   - Are you very active around the house?
   - If you are employed, is your work active?
2. How active was your child before the first lockdown?
   - Did your child use active modes of transport to school?
   - Did they participate in any active clubs or hobbies?
   - If you feel you can answer, were they active during playtimes?

***First school closure/lockdown 1 (March 2020)***

1. Did your child attend school at this time? (i.e. parents were key workers)
   1. If so, to what extent do you feel this influenced their activity levels?
2. To what extent do you feel the first lockdown/school closure influence your activity levels?
3. How about your child’s?
4. What were the key factors that influenced any changes to your/your child’s PA at this time?

Pool of prompts (exact prompts used will be guided by the contact’s role/previous discussions):

| **Policy** | |
| --- | --- |
| 1. To what extent do you feel that any policy/legal restrictions associated with COVID-19 effected your/your child’s (i.e. lockdowns, social distancing)? | □ |
| **Environment** | |
| 1. To what extent did you/your child’s opportunities to be physically active outside of school change at this time?    1. How did you feel about these changes? | □ |
| 1. How do you feel any changes to school or school curriculum PE influence your child’s activity levels?    1. How did you feel about these changes?    2. Did you feel supported in doing PE from home? | □ |
| **Strategies to promote PA** | |
| 1. To what extent did you feel supported and encouraged by the school or other organisations to be physically active at this time? | □ |
| **Attitudes** | |
| 1. Can describe your attitude and thoughts towards physical activity at this time? Had it changed? | □ |
| 1. To what extent do you feel that the school’s attitude towards PE and other physical activities changed at this time? | □ |
| 1. To what extent do you feel that being less or being more active was the norm during lockdowns, or did it stay the same? Was this the same for both child and parent? | □ |
| **Social** | |
| 1. To what extent do you feel your child’s active play with their friends changed at this time? | □ |
| 1. To what extent did social distancing and not being able to see other people influence your/your child’s physical activity? | □ |
| **Motivation** | |
| 1. To what extent do you feel that your/child’s motivation for physical activity changed at this time? | □ |
| 1. Have you noticed changes related to confidence in your ability to do PA in your child? If so, how did it change? | □ |
| Emotion | |
| 1. Thinking back to how you felt at this time, to what extent did your/your child’s feelings influence your activity levels? For example, was lockdown 1 a novelty that made you want to be more active or were you very worried and did not want to leave your house? | □ |

***First return to school (Autumn 2020)***

1. Did your child attend school this time? If so, what had changed?
2. To what extent did the lifting of the lockdown and return to school influence your physical activity levels?
3. How about your child’s?
4. What were the key factors that influenced any changes to your/your child’s activity at this time?

***Second school closure (January – March 2021)***

1. Did your child attend school at all at this time?
   1. If so, what had changed?
   2. To what extent do you feel this influenced their activity levels?
2. Thinking back to the first lockdown/school closure, to what extent did your/your child’s activity levels differ in the second lockdown/closure compared to the first?
3. Can you please describe any differences between this lockdown/school closure and the first?

***Second (most recent) reopening of schools (April – July 2021)***

1. To what extent did the most recent return to school influence your activity levels?
2. How about your child’s?
3. What were the key factors that influenced any changes to your/your child’s PA at this time?

***Other factors influencing PA***

1. Are there any other factors that we haven’t discussed that you think have influenced your/your child’s activity levels over the course of the pandemic so far?

***Electronic device use/screen time***

1. How has your child’s use of electronic devices changed along the way – total time and how they use them (i.e. for school work/socializing/gaming or watching things)?

**Current solution-focused ideas for themselves and their child**

1. Evidence from government reports and other research suggests that physical activity has decreased since the pandemic started. To what extent does this match up with your experiences?
2. Are there any barriers that are ***currently*** preventing you/your child from being more active?
   1. How and who could support you to remove these barriers?
3. Is there anything that could help to boost your/your child’s motivation to be active?
4. Is anything needed to help enable you/your child to do more of the active things you/they wish to do?
   1. Where/when/how should it be available?
   2. How could the school support it?
   3. How could local communities/local government support it?
   4. How could national government support?

**Closing statement**

- Is there anything else you’d like to tell us about the things we talked about today?
- Do you have any questions for me?
- We appreciate you sharing your thoughts and opinions with us!

1. **School staff interview guide**

**Introduction**

- Thank participant for taking part in the interview and introduce myself
- Describe the purpose of the ACTIVE-6 project and the interview
- Discuss definition of PA
- Key components of the interview
  - Discuss any changes within the school that may have influenced PA throughout different phases of the pandemic
  - Your thoughts on how these changes may have influenced levels of physical activity among Year 6 children
  - The approach of the school to promote physical activity during and after national school closures
  - Whether there are any key lessons or suggestions for the future
- Emphasise that there are no wrong answers and that I am not here to judge, but to explore their experiences related to what changed and how this may have impacted PA among Year 6 children.
- Confidentiality procedures
  - I’d like to tell you that I will be **recording the conversation** to help me remember what you said.
  - You can ask for the recording to be stopped at any time.
  - After the interview, the recording will be transcribed/written up word for word
  - After the interview has been transcribed, the audio recordings will be destroyed
  - In the transcripts, all identifying information will be removed, so nothing you say can be connected to you in any way
  - Anonymised transcripts will be held safely and securely for 20 years
  - Please remember that you can interrupt the interview at any point if you need to and that you are free to withdraw your data from this study up to three weeks after the interview. You do not need a reason for withdrawing.
  - You are also completely free to not answer any of the questions if you do not want to you
  - Do you have any questions for me?
  - Are you happy to proceed with the interview? ***If yes, begin the recording***
- For the recording, can you please confirm that you have been given information explaining about the study and that you understand what this project is about?
- Can you please confirm that you have had the opportunity to ask questions and discuss this study?
- Can you please confirm that you are aware that the interview data (transcriptions) will be stored anonymously and securely for 20 years?
- Can you please confirm that you are aware that you are free to withdraw your data from the study at any time up to three weeks after this interview has taken place **[X DATE]**, and that you do not need to give a reason for withdrawing?
- And finally, can you please confirm that you are happy to take part in the interview?

**Icebreaker/Demographics questions**

The first few questions will help me get to know you a little better, what your role is in the school, and your thoughts about PA.

1. Please tell me what role you hold in the school and what level of influence you have over school policies surrounding physical activity.
2. Are you a qualified teacher? If so, how long have you been teaching?
3. Does your role involve teaching PE? (If so how often?)
4. What kind of involvement do you have with the extracurricular programme at your school?
5. What personal value do you place on physical activity?

**Changes in/factors influencing physical activity among year 6 pupils**

The next set of questions discuss any differences/changes in the school and how these may have influenced PA among Year 6 children over the course of the pandemic so far.

***Pre-COVID 19***

1. How active would you say Year 6 pupils were at school before the pandemic?
   1. How active were playtimes, generally?
   2. What was participation in active after-school clubs like? An active after-school club is a club at your school that is all about playing a sport or being active.
   3. How frequently did pupils use active modes of travel to get to and from the school? (*e.g.* walking, cycling, scooting)

***First school closure (mid March)***

1. Can you describe any differences/changes within the school that might have influenced physical activity among Year 6 pupils at this time?

Pool of prompts (exact prompts used will be guided by the contact’s role/previous discussions):

| **Policy** | |
| --- | --- |
| 1. Can you describe any PA-related school policy changes at this time?    1. What did you think about these policy changes?    2. To what extent did you feel supported by the school/government in implementing these policies?    3. Can you outline any funding changes/problems that influenced school PA at this time?    4. How did you feel about these changes? | □ |
| **Environment** | |
| 1. To what extent did opportunities for PA within the curriculum change?    1. What did you think about these changes? | □ |
| 1. To what extent did extracurricular opportunities for PA change?    1. What did you think about these changes? | □ |
| 1. To what extent do you feel that staff were supported in delivering PA at this time? | □ |
| **Strategies to promote PA** | |
| 1. Can you outline any strategies your school created to promote PA during school closures?    1. Can you describe the decision making/design process for these strategies? What did you think about them?    2. What were your experiences/thoughts of these strategies? How easy/difficult was it?    3. How effective do you think they were in achieving their aims?    4. To what extent would you change any of these strategies? | □ |
| **Attitudes** | |
| 1. To what extent do you feel that any attitudes towards PA within the school changed at this time? | □ |
| 1. To what extent do you feel that any attitudes of parents towards PA changed at this time? | □ |
| 1. Can you describe the school’s priorities during the first school closure and their relation to school physical activity? | □ |
| **Social** | |
| 1. To what extent do you feel active play among pupils changed at this time? | □ |
| **Motivation** | |
| 1. To what extent do you feel that motivation for PA changed within the school this time? (i.e. among pupils or school delivering PA) | □ |
| 1. To what extent do you feel confidence in ability to do PA changed among Year 6 pupils at this time? | □ |

1. If you feel you can, can you comment on the implication of these changes for overall physical activity levels among Year 6 pupils? (Frequency or type differences?)
2. How many children attended school in-person during the first school closure? (How many Year 6 – approx.)
3. To what extent do you think attending school in-person influenced their levels of school-related PA?

***First return to school (Autumn 2020)***

1. Can you describe any differences/changes within the school that may have influenced PA among Year 6 pupils following the first return to school?

| **Environment** | |
| --- | --- |
| 1. To what extent do you feel that any COVID restrictions in the school influences activity levels?    1. Bubbles    2. Restrictions on movement    3. Restriction on play equipment    4. Spaced out desks    5. Outdoor learning | □ |

1. If you feel you can, can you comment on the implication of these changes for overall physical activity levels among Year 6 pupils?

***Second school closure (January - March 2021)***

1. Thinking back to any differences/changes within the school that impacted PA during the first closure, to what extent did any changes during the second closure differ to the first?
2. Can you describe any differences between this school closure and the first?
3. To what extent do you feel that activity levels among Year 6 pupils differed during this school closure compared to the first?
4. Can you tell me about any differences in the number of children attending school in-person during the second school closure compared to the first?
5. To what extent do you feel attending school in-person influenced their levels of school-related PA?

***Second/most recent return to school (April 2021)***

1. Can you describe any changes within the school after the return to school in April that may have influenced physical activity among Year 6 pupils?
2. If you feel you can, can you comment on the impact of these changes might have had on overall physical activity levels among Year 6 pupils?
3. Looking back over the course of the pandemic, to what extent did your role differ and change in relation to physical activity?
   1. How about the role of other staff members?

***Other factors influencing PA among year 6 pupils***

1. Are there any other factors that we haven’t discussed that you think have influenced or changed levels of PA among Year 6 pupils throughout the course of the pandemic so far?

**Key lessons and recommendations related to encouraging PA among pupils during a pandemic**

The final set of questions explore your thoughts on any lessons learned and future recommendations.

1. Can you describe any key lessons the school learned related to encouraging PA among year 6 pupils from the experience gained during the course of the pandemic so far?
2. To what extent do you plan to continue using any changes/strategies related to PA made during the pandemic?
3. To what extent do you think anything could have been helpful when schools first closed for parents or staff to help promote PA?
4. If we were put in a similar situation again, is there anything you think you/your school would have done differently in terms of supporting pupil physical activity

**Closing statement**

- Is there anything else you’d like to tell us about what we have talked about today?
- Do you have any questions for me?
- Thank participant and end interview.

1. **Student focus group guide**

**Introduction (10-15 minutes)**

- Thank children for participating in the ACTIVE-6 project
- Introduce myself
- Explain why this project is important and what it means for them

***Active travel (10 minutes)***

1. Who here has ever walked, biked, or scooted to school before?
2. Who has changed how they travel to school since coming back after lockdown?
3. If you could choose anyway to travel to school, what would you choose? Why? What is stops you from doing that?

**Physical activity during school time (15 minutes)**

***School PE***

1. What is everyone’s favourite thing to do in PE? What did you like about it?
2. How did everyone feel about PE before, during, and after?
3. Has your feelings changed toward PE since after the lockdowns?

***Breaktime***

1. Who likes playing in the playground? What kind of things do you do?
2. Did anyone miss not being able to play in the playground when schools were closed?
3. How was it going back into the playground after lockdown?
4. Did it feel different?

**Physical activity outside of/after school (15 minutes)**

***Activity***: Ask children to draw themselves doing the physical activity they do the most at home. For example, playing with friends and going on walks with family. Ask them to add words around it to describe how it makes them feel. Children will have 2 minutes to draw their best stick figure-style drawing.

***Active clubs***

1. Who did an active club, such as football or swimming, before coronavirus? **(SHORT)**
2. After schools were closed, what happened to your active club? Did it continue online or was it cancelled?
3. What was it like going back to active clubs after lockdown?
4. Did anybody choose not to go back to their active club because of COVID?
5. Who has started an active club after coming back to school? What made you want to start one?

***At home/screen time***

1. How did everyone spend their free time during lockdown?
2. Was this different to what you used to do before lockdown?
3. Are you still spending your free time that way?
4. Does anyone think their parents rules about screen time changed?

**Closing statement and questions**

- Thank all the children and tell them they have been a big help with our research
- Overall, is there anything anyone would like to talk about or mention that we haven’t already discussed?
- Are there any questions?
- Say goodbye and end focus group

**Wave 2 – February to July 2022**

1. **Parent interview guide**

**Introduction**

Thank you for agreeing to take part in this interview, your views and opinions are really important to us. In the interview today, I would like to talk about three main points:

- Whether there are any differences in your and your child’s pre-pandemic and recent physical activity patterns
- Factors you feel have influenced any changes in physical activity patterns
- Whether you think there is anything that can be done to help support you and your child’s physical activity

We are really interested in your honest opinions, we are not here to judge you, and we do not want you to feel like you should answer any of the questions in a certain way. There are no right or wrong answers, and as much detail you can give on the topics as possible is really appreciated.

*Confirm participant has read the information sheet/consent form and are still happy to participate. Gain verbal consent if participant has not consented during the Active-6 sign up process. If the participant is happy to proceed, the recording will start.*

**Changes in activity patterns**

***Parents***

1. Do you have any favourite physical activities? Why are they your favourite?
2. How would you describe your activity levels before the first lockdown (pre-March 2020)
   - Did you often use active modes of transport?
   - Were you part of any active clubs or have any active hobbies?
   - Are you active around the house?
   - If you are employed, is your work active?
3. To what extent do you feel that your current activity levels are different to pre-pandemic levels?
4. Can you describe the key factors that have influenced any changes to your activity patterns?

Prompts:

1. Priorities
2. Motivation
3. Discovering new types of activity in lockdown
4. COVID fears and worries
5. *Changes to family physical activity patterns*
6. In the first part of the Active-6 project, we found that, generally speaking, during the last half of 2021 parents were ***similarly*** active than a similar sample of Year 6 parents from 2018 (pre-pandemic). Does this finding match your experience, either personal or of those around you? Why do you think this may or may not be the case?
7. To what extent do you feel that any changes to your activity patterns are likely to last long-term or change in the future?
   - Did your activity levels stay consistent throughout the pandemic? Or did you make an effort to increase/return to similar levels following the easing of restriction?

***Year 6 Child***

1. Does your Year 6 child have any favourite physical activities? Why do you think it’s their favourite?
2. How active was your child before the first lockdown (pre-March 2020)?
   1. Did your child use active modes of transport to school?
   2. Did they participate in any active clubs or hobbies?
   3. Does your child do any types of active play?
3. To what extent do you feel that your Year 6 child’s current activity levels are different to pre-pandemic levels?
4. Can you describe the key factors that have influenced any changes to your child’s activity patterns?

Prompts:

1. Active club provision/interest
2. Motivation
3. Confidence/self-efficacy
4. Changes to active play outside of school
5. Changes to child’s hobbies/interests
6. Conversely to what we found with parents, we observed that, generally speaking, during the last half of 2021 Year 6 children were ***less*** active than a similar sample of Year 6 children from 2018. Does this finding match your experience, either personal or of those around you? Why do you think this may or may not be the case?
7. To what extent do you feel that any changes to their activity patterns are likely to last long-term or change into the future?

***Screen time/electronic device use***

1. How would you describe your child’s current screen time/device use?
2. Has the way they use devices changed at all?
3. Do you feel that screen time/device use impacts physical activity levels?

Prompts:

- Have you ever changed the amount of leisure screen time your child is allowed because of increased online school work?
- How do you view TV vs phones/tablets/games consoles? Do you consider them the same?

***Solution focused ideas***

1. Are there any barriers that are ***currently*** preventing you/your child from being more active? How and who could support you to remove these barriers?
2. Is anything needed to help you and/or your child engage in more:
   - Active travel (*i.e.* walking to school)
   - Active clubs at school (*i.e.* school sports teams)
   - Active clubs outside of school (*i.e.* community sports teams, scouts/brownies)
   - Active play outside of school (*i.e.* playing football with friends, cycling, tag etc.)
   - Family physical activities (*i.e.* family walks, family cycling, family swimming)

**Closing statement**

- Is there anything else you’d like to tell us about the things we talked about today?
- Do you have any questions for me?
- We appreciate you sharing your thoughts and opinions with us!

1. **School contact interview guide**

**Introduction**

Thank you for agreeing to take part in this interview, your views and opinions are really important to us. In the interview today, I would like to talk about three main points:

- Current physical activity patterns among Year 6s
- Whether there are any lasting changes since the pandemic within the school, and the extent to which you feel these impact physical activity among Year 6 children
- What kind of support could be provided to schools to help improve physical activity provision

We are really interested in your honest opinions, we are not here to judge you, and we do not want you to feel like you should answer any of the questions in a certain way. There are no right or wrong answers, and as much detail you can give on the topics as possible is really appreciated.

*Confirm participant has read the information sheet/consent form and are still happy to participate. Gain verbal consent if participant has not completed and returned their school contact consent form. If the participant has consented and is happy to proceed, the recording will start.*

**Child physical activity levels in school**

1. How active would you describe the ***current*** activity levels/patterns of the Year 6 pupils?
   1. How active are playtimes, generally?
   2. What is participation in active after-school clubs like? An active after-school club is a club at your school that is all about playing a sport or being active.
   3. How frequently do pupils use active modes of travel to get to and from the school? (*e.g.* walking, cycling, scooting)
   4. Are these physical activity patterns/levels different than before the pandemic?
2. Using the activity belt data we collected last year, we observed that, generally speaking, during the last half of 2021 Year 6 children were ***less*** active than a similar sample of Year 6 children from 2018.
   1. What do you think about this?
   2. Does this finding match your experience, either personal or of those around you?
   3. Why do you think this may or may not be the case?
3. What do you feel are the factors influencing any differences?

**Lasting structural changes within the school and their impact on physical activity**

1. After speaking with members of school staff last year, there was a lot of discussion surrounding differences/changes within the school due to the pandemic. Can you describe any differences that are still apparent within the school?
   1. Funding changes
   2. Restrictions on play equipment/facilities
   3. Bubbles/pods
   4. After school club provision
   5. Reductions in interschool sports/events
   6. Academic priorities
   7. Outdoor/active learning
2. To what extent do you feel that any changes are impacting physical activity among Year 6s?
3. To what extent do you anticipate any lasting changes within the school continuing in the future?

**Lasting changes within children**

1. Parents, children, and school staff last year spoke of changes within children, such as motivation or weight gain, that they had observed or experienced after the most recent reopening/easing of lockdown in April 2021. To what extent do you feel that any changes within children are still apparent at the moment?
   1. Emotional overwhelming and fatigue
   2. Social conflict/challenges during play/breaktimes
   3. Weight gain and loss of fitness
   4. Active club interest/participation
2. To what extent do you feel these changes are likely to continue in the future?

**Solution focused ideas**

One of our primary goals of the Active-6 project is to provide information to policy makers on what can be done to help support physical activity among children. As part of this, we are very interested in what you, and other members of staff, feel could be done to support physical activity at school. Your thoughts and opinions are really important to us. Please consider the following questions:

1. Do you feel there is anything needed to help Year 6 children engage in more:
   1. Active travel (*i.e.* walking to school)
   2. Active afterschool clubs (*i.e.* school sports teams)
   3. Active clubs outside of school (*i.e.* community sports teams, scouts/brownies)
   4. Active play during breaktimes or after school (*i.e.* playing football with friends, tag etc.)
2. Can you describe any challenges that you or other members of staff experience related to physical activity provision at school?
3. What do you and other members of staff feel could be done to support physical activity in school?
4. How does your school currently spend physical activity-related funding?
5. To what extent do you feel that funding for physical activity in school is sufficient?
6. If you are able to comment, to what extent do you feel the school’s use of funding (*i.e.* sport premium) is utilised effectively and appropriately?
7. If the school was provided with additional funding for physical activity and PE, how might you spend it?
8. How would you describe the school current afterschool club provision? What could be done to support/improve afterschool clubs?
9. To what extent do you feel the senior leadership team has an important role in physical activity in school?
10. To what extent do you feel teachers are supported in promoting physical activity?
11. To what extent do you agree/disagree that academic or other pressures negatively impact physical activity among Year 6s?
12. To what extent do you feel that teacher training is sufficient to deliver good physical activity/PE to children?
13. To what extent do you feel that the current PE curriculum is effective and engaging for Year 6 children? How do you think it could be improved?
14. To what extent do you physical activity and PE are prioritised at school?

**Closing statement**

- Is there anything else you’d like to tell us about the things we talked about today?
- Do you have any questions for me?
- We will be sharing the results with all participants once the study is complete.
- We appreciate you sharing your thoughts and opinions with us!

1. **Focus group plan and topic guide**

**Introduction**

Thank children for participating in the ACTIVE-6 project

- Introduce myself
- Explain why this project is important and what it means for them

***Activity 1: Making name tags***

- All children and focus group facilitators make name tags.

Give overview of focus group format:

- - We want to hear about your physical activity and how COVID-19 might have affected it
  - My role – I will be here to guide the discussion, but the discussion will mainly be among you all. Please feel free to ask each other questions and share your thoughts on what others say.
  - You are all the experts, so we really need your help!
  - Does everybody understand so far and does anyone have any questions?

***Activity 2: Hot potato***

Active game to discuss and conceptualise definition of physical activity. Ask the group to stand in a circle. Children then pass a ball (moon ball) or frisbee to the child on their left. When a child catches a ball they need to shout out a physical activity (*i.e.* football, walking to school). Repeating activities are not allowed. Child needs to put a hand behind their back if they cannot give a new answer. I will also participate to give examples of physical activities that might not be as obvious and help clarify the definition.

- Ethical procedures
  - If it is ok with you all, I will be **recording the conversation** to help me remember what we all talked about.
  - Show the microphone to the group
  - Someone will be writing this up word for word, so it is really important not to talk over each other so that the recording is clear
  - Your names and information will be completely removed from the write up, so no one can know who said what.
  - If you would like to leave at any time, that is perfectly fine, so please just let me know.
  - Does anyone have any questions for me?
  - Would anyone like to use the bathroom before we start?
- Is everyone happy to take part in this discussion?
- Is everyone happy for us to start? **If yes, begin the recording**

**Focus group discussion**

***Active travel***

1. How does everyone normally travel to and from school? **(keep brief)**
2. Have you always travelled to school and home that way, or has it changed?

Prompts:

- If they could, would anyone change the way they travel to and from school?
- Does anyone feel the way they travel to school now is different because of COVID?

**Physical activity during school time**

1. What is everyone’s favourite thing to do in PE? What do you like about it? **(keep brief)**

***Activity 3: Design your ideal PE lesson***

Working in groups of 2-3, the children will design their ideal PE session using whiteboards. These will then be used to facilitate discussions surrounding what might have changed and what could be done to promote physical activity. The groups will then take it in turns to describe their ideal lesson

Prompts:

- What do you think of your current PE lessons?
- How is your ideal PE lesson different or the same as your actual lesson?
- Have your PE lessons changed since COVID?

1. What kind of things do you all usually do at breaktimes? **(keep brief)**

***Activity 4: Design your ideal breaktime***

Working in groups of 2-3, the children will design their ideal breaktime using whiteboards. These will then be used to facilitate discussions surrounding what might have changed and what could be done to promote physical activity. The groups will then take it in turns to describe their ideal lesson

Prompts:

- What do you think of breaktimes at the moment?
- How is your ideal breaktime different or the same as your actual breaktime?
- Have your breaktimes changed since COVID?

**Physical activity outside of/after school**

***Active clubs***

Discuss definition of active clubs as “any organised group activity outside of school where you move your body, sometimes get out of breath, and heart beats a bit faster”, drawing on examples used during the hot potato activity – e.g. cubs/scouts too, not just sports.

1. What kinds of organised activities does everyone do after school? **(keep brief)**
2. When did everyone go back to their active club?
3. If at all, how have your clubs changed since COVID?
   1. Smaller groups
   2. Location
   3. Children/friends who attended
   4. Rules/restrictions
   5. Has anybody not gone back to the active club they did before COVID? Can you describe the reasons?
4. What kind of things does an active club need to have that would make you want to go?
   1. Friends attending
   2. What makes you feel comfortable/uncomfortable
5. Does anyone feel that the number of active clubs they do has changed?
   1. Has anybody recently started a new active club? What has that been like?
   2. Is anyone still not able to do the active club they would like to because of COVID, or another reason?
6. *Does anyone feel that there are certain clubs that are too expensive?*
   1. *Expensive kit*
   2. *Expensive travel*
   3. *Expensive cost*
7. *Can anyone describe a fun and cheap physical activity?*

***At home/screen time***

1. How does everyone spend their free time after school and at the weekends (outside of clubs)?

Prompts:

- 1. Who are you with?
  2. Spending time outdoors
  3. Playing outside alone or with others
  4. Play at home – crafts, baking, games etc
  5. Playing with friends or family online
  6. Watching TV or using electronic devices
  7. School work/study
  8. Other non-active activities

1. Who feels that the way they spend free time outside of school has changed since COVID?
   1. Would anybody like to play more in person more, but feel like they can’t? Why is this?
2. Does anyone feel their screen time or device use is different since COVID?
   1. Do you feel this impacts the amount of other activities that you do, such as playing outside?
   2. Can anyone describe their parent’s rules about screentime? What do you think about them?
   3. Do you think your parent’s rules about screen time have changed since the pandemic?
   4. Do you feel that using screens for schoolwork and screens for fun are the same or different?

**Exploring previous qualitative findings**

1. Did anyone feel more tired or find it difficult after COVID when everything like clubs and school reopened? How is this for you at the moment?
2. Did anyone find being around lots of people again after lockdown difficult, how are those feelings for you now?
3. Last year, I spoke to similar groups of year 6 children just before Christmas. These children spoke of not wanting to do as much physical activity as they did before COVID. They told me this was because they felt too tired because they had lost their fitness in lockdown and because of going back to school after lockdown and being surrounded by lots of other children.
4. What does everyone think of this?
5. Does anyone still feel this way, or did anyone feel this way at first but now feels better?
6. What made it feel better?
7. Would there be anything that could/ could have helped make it feel better?

**Closing statement and questions**

- Thank all the children and tell them they have been a big help with our research
- Overall, is there anything anyone would like to talk about or mention that we haven’t already discussed?
- Are there any questions?
- Say goodbye and end focus group
